# Identification and Characterization of Germ Cell Genes Expressed in the F9 Testicular Teratoma Stem Cell Line

**DOI:** 10.1371/journal.pone.0103837

**Published:** 2014-08-25

**Authors:** Jun Tae Kwon, Sora Jin, Heejin Choi, Jihye Kim, Juri Jeong, Jaehwan Kim, Youil Kim, Byung-Nam Cho, Chunghee Cho

**Affiliations:** 1 School of Life Sciences, Gwangju Institute of Science and Technology, Gwangju, Korea; 2 Department of Biology, The Catholic University of Korea, Bucheon, Korea; Clermont Université, France

## Abstract

The F9 cell line, which was derived from a mouse testicular teratoma that originated from pluripotent germ cells, has been used as a model for differentiation. However, it is largely unknown whether F9 cells possess the characteristics of male germ cells. In the present study, we investigated spermatogenic stage- and cell type-specific gene expression in F9 cells. Analysis of previous microarray data showed that a large number of stage-regulated germ cell genes are expressed in F9 cells. Specifically, genes that are prominently expressed in spermatogonia and have transcriptional regulatory functions appear to be enriched in F9 cells. Our *in silico* and *in vitro* analyses identified several germ cell-specific or -predominant genes that are expressed in F9 cells. Among them, strong promoter activities were observed in the regions upstream of the spermatogonial genes, *Dmrt1* (doublesex and mab-3 related transcription factor 1), *Stra8* (stimulated by retinoic acid gene 8) and *Tex13* (testis expressed gene 13), in F9 cells. A detailed analysis of the *Tex13* promoter allowed us to identify an enhancer and a region that is implicated in germ cell-specificity. We also found that *Tex13* expression is regulated by DNA methylation. Finally, analysis of GFP (green fluorescent protein) TEX13 localization revealed that the protein distributes heterogeneously in the cytoplasm and nucleus, suggesting that TEX13 shuttles between these two compartments. Taken together, our results demonstrate that F9 cells express numerous spermatogonial genes and could be used for transcriptional studies focusing on such genes. As an example of this, we use F9 cells to provide comprehensive expressional information about *Tex13*, and report that this gene appears to encode a germ cell-specific protein that functions in the nucleus during early spermatogenesis.

## Introduction

Male germ cell development, or spermatogenesis, is a complex process that involves successive mitotic, meiotic, and post-meiotic phases [1], [2]. The tightly regulated nature of this process, which occurs in the seminiferous tubules of testes, indicates that a highly organized network of genes is expressed in germ cells during spermatogenesis. Three levels of control regulate gene expression during spermatogenesis: intrinsic, interactive and extrinsic control [3]. The intrinsic program determines which genes are utilized and when these genes are expressed. The unique feature of this program is germ cell- and stage-specific gene expression. The interactive process, which involves crosstalk between germ cells and somatic cells, is essential for germ cell proliferation and progression. Finally, extrinsic influences, including steroid and peptide hormones, regulate the interactive process. Not all extrinsic regulation act through Sertoli cells. Retinoic acid (RA), the active derivative of vitamin A, is essential for spermatogenesis and has direct effects on germ cell development [4].

Pluripotent/primordial germ cells (PGCs), which colonize the developing gonads by active migration [5], [6], turn into gonocytes. The latter differentiate into spermatogonial stem cells (SSCs), which provide a continuous supply of differentiating spermatogonia during spermatogenesis [7]. Signaling and transcriptional regulation are crucial for germ cell pluripotency, survival and differentiation. Misregulation of germ cell pluripotency may lead to tumor formation [8]. Testicular teratomas originate from primordial germ cells [9]. Although teratomas are tumor, they possess the capacity to produce all three germ cell layers. Therefore, testicular teratomas provide a useful tool for investigating the intersection of pluripotency, differentiation and tumorigenesis.

The F9 cell line was isolated as a subline of a testicular teratoma (designated OTT6050) that was established by implanting a six-day-old embryo in the testis of a 129/J mouse [10]. The F9 cell line is a nullipotent cell line that is unable to undergo spontaneous differentiation. However, F9 cells can differentiate into endodermal-like derivatives following treatment with several agents, including retinoic acid. Therefore, the F9 cell line has been used as a model for analyzing the molecular mechanisms of differentiation [11].

Because F9 cells originate from embryonic cells containing primordial germ cells, it is possible that the intrinsic genetic program involving spermatogenic stage- or cell-specific genes is partially active in F9 cells. If this is the case, the F9 cell line could be used as an alternative cell line for investigating germ cell transcription. In fact, the testis-specific promoter region of the human pituitary adenylate cyclase-activating polypeptide gene (*PACAP*) was previously investigated using F9 cells [12]. To address this issue, we herein used microarray data to analyze the transcripts expressed in F9 cells. We found for the first time that a large number of stage-specific germ cell genes are expressed in F9 cells. Various expressional analyses and reporter assays showed that F9 cells can be used for transcriptional studies of genes that are stage-specifically expressed in early male germ cells. In particular, genes encoding doublesex and mab-3 related transcription factor 1 (*Dmrt1*), stimulated by retinoic acid gene 8 (*Stra8*) and testis expressed gene 13 (*Tex13*) were significantly expressed in F9 cells. Since the functions and transcriptional mechanisms were previously reported for *Dmrt1* and *Stra8* [35]–[40], we focused on the expression of *Tex13* in F9 cells. Our comprehensive analysis of the *Tex13* promoter allowed us to identify regions responsible for the germ cell specificity and strong enhancer activity of this promoter. Moreover, *Tex13* promoter showed cell-type specific DNA methylation. In addition, we found that *Tex13* encodes a potential nucleocytoplasmic shuttling protein. Our study is the first comprehensive and systematic investigation of germ cell genes expressed in F9 cells.

## Materials and Methods

### Microarray data analysis

We obtained microarray data representing spermatogenic cells, F9 cells and J1 embryonic stem cells from the Gene Expression Omnibus (GEO) (http://www.ncbi.nlm.nih.gov/gds/). The GSE4193 dataset contained expression profiles obtained from a purified population of spermatogenic cells [13]; the GSE31280 dataset contained the gene expression profile of F9 cells [14]; and the GSE9978 dataset contained array data obtained from J1 embryonic stem cells [15]. Feature-level data (CEL) files were downloaded and imported into R program for normalization. R is an open source statistical scripting language (http://www.r-project.org). All expressional data were normalized using the GCRMA method [16]. Expressional data obtained from spermatogenic cells (spermatogonia, spermatocytes and spermatids), F9 cells and J1 cells were combined into a microarray dataset. The combined array data were normalized by quantile normalization using the “normalize.quantiles” function from R/Bioconductor package. The averages between duplicates derived for each sample were calculated. For each experimental group (Spermatogonia-F9, Spermatocyte-F9 and Spermatid-F9), genes with absolute fold changes greater than 1.5 were chosen as differentially expressed genes (DEGs) and subsequently analyzed using the DAVID Functional Annotation Tool for gene ontology (GO) (http://david.abcc.ncifcrf.gov/) [17]. A functional annotation chart is useful for identifying annotation terms that are enriched in the submitted gene list; a smaller *p*-value indicates increasing significance of the GO term, and fold enrichments of 1.5 and above are considered interesting.

### Reverse transcription PCR

To validate our analysis of the available microarray results, we performed reverse-transcription PCR (RT-PCR) using total RNA from testis, F9 cells and NIH3T3 cells. Total RNA was extracted using the TRIzol reagent (Molecular Research Center) according to the manufacturer's protocol, and cDNA was synthesized by random hexamer and oligo(dT) primers using the Omniscript reverse transcriptase (Qiagen). The utilized gene-specific primers are listed in Table S1. PCR was performed for 30 cycles of 94°C for 30 s, 55°C for 30 s, and 72°C for 1 min 20 s. Glyceraldehyde-3-phosphate dehydrogenase (*Gapdh*) was amplified as a control (forward, 5′-TGA AGG TCG GAG TCA ACG GAT TTG GT-3′ and reverse, 5′-CAT GTG GGC CAT GAG GTC CAC CAC-3′). The testis-specific expression of the nine tested genes was examined by RT-PCR in eight different mouse tissues (testis, ovary, brain, heart, kidney, lung, liver, and spleen). Specific expression at different stages of spermatogenesis was established using total RNA obtained from testes of prepubertal and adult male mice (ages 8, 10, 12, 14, 16, 20, 30, and 56 days), and from the testes of *W/W* ^v^ mutant mice, which lack germ cells. All animal investigations were carried out according to the guidelines of the Animal Care and Use of Gwangju Institute of Science and Technology. The protocol was approved by the Animal Care and Use Committee of Gwangju Institute of Science and Technology (Permit number: GIST 2011–13).

### Cell culture

F9 (CRL-1720) and NIH3T3 cells (CRL-1658) were obtained from the American Type Culture Collection (ATCC, Manassas, VA). Cells were maintained in Dulbecco's modified Eagle's medium (DMEM) supplemented with 10% fetal bovine serum (F9) or 10% bovine caput serum (NIH3T3), along with 2 mM glutamine, 100 U/ml penicillin and 10 mg/ml streptomycin. The culture vessels for F9 cells were coated with 0.1% gelatin prior to use. Since RA is known to induce the differentiation of F9 cells, the chemical was not included in the media during cultivation. The cells were maintained in a humidified 5% CO_2_ atmosphere at 37°C, the cell media were changed every 1–2 days, and the cells were subjected to passage every 3–4 days.

### Generation of reporter gene constructs

A luciferase reporter assay system (Promega) was used to measure promoter activity. To construct the 1.5-kb promoter luciferase reporter plasmid for five genes (*Dmrt1*, *Stra8*, *Tex13*, *Triml1* and 1700061G19Rik), DNA fragments corresponding to the putative promoters predicted by DBTSS (http://dbtss.hgc.jp./) were prepared by PCR using the pfu DNA polymerase (Enzynomics) with mouse genomic DNA isolated using Dneasy Blood & Tissue kit (Qiagen). The utilized primers are listed in Table S1. Several deleted versions of the *Tex13*-luciferase reporter plasmids were also prepared. The sequence of each PCR product was confirmed by DNA sequencing (Macrogen), and each fragment of interest was cloned into the multi-cloning site of the promoter-less pGL3-Basic (Promega) plasmid.

### DNA transfection and luciferase assay

Cells were plated to 24-well plates at 1.5×10^5^ cells/plate. After 24 h, when cells were at 50–60% confluence, the Lipofectamine LTX transfection reagent (Invitrogen) was used to transfect 400 ng of the indicated DNA construct together with 8 ng of pRL-TK (Promega), which contains the Renilla luciferase gene driven by the SV40 promoter, and was used an internal control for normalization of transfection efficiency. Twenty-four hours later, whole cell extracts from triplicate wells were assayed. Lysates were prepared using 100 µl of passive lysis buffer (Promega), and luciferase activity was determined from 30 µl of each lysate using the Luciferase Reporter Assay (Promega) and a Centro LB 960 DLReady Micro Plate Illuminometer (Berthold Technologies). Each experiment was repeated at least three times. The obtained promoter activity was normalized against that of the SV40 promoter, and was reported as the fold activity compared to that from pGL3-Basic.

### Purification of genomic DNA and bisulfite methylation assay

Genomic DNA (gDNA) was purified from F9 cells and NIH3T3 cells using a DNeasy blood and tissue kit (Qiagen) according to the manufacturer's protocol. Promoter methylation was assessed by bisulfite sequencing. In brief, cytosine-to-uracil conversion was performed on 1.0 µg of gDNA using an EZ DNA Methylation-Gold kit (Zymo Research) according to the manufacturer's provided protocol. PCR reactions were performed on the converted DNA with promoter specific-primers designed using the online MethPrimer software (http://www.urogene.org/methprimer/). PCR was performed for 48 cycles of 95°C for 30 s, 48°C for 30 s, and 72°C for 30 s. For bisulfite sequencing, the amplified PCR products were cloned into the pTOP v2 vector (Macrogen). From each cell line (F9 and NIH3T3) a total of 10 independent clones containing each of the desired PCR products were sequenced.

### *In vitro* methylation

The −402/+20 *Tex13* promoter was inserted into pGL3-Basic, and the vector was incubated at 37°C for 4 h with the methyltransferase enzyme, *M.Sss*I (NEB) in the presence of the methyl group donor S-adenosylmethionine (SAM) (NEB). Mock-methylated plasmids were incubated without enzyme but in the presence of SAM. Samples were purified using a PCR purification kit (LaboPass). The mock-methylated and methylated plasmids were subjected to diagnostic digests with *Hpa*II, a methylation-sensitive enzyme, to confirm the efficacy of the *in vitro* methylation.

### Localization of recombinant TEX13 in F9 cells

The coding region of mouse *Tex13* (NM_031381 in GenBank) was amplified by RT-PCR and subcloned into the N terminus of pEGFP-N2 (Clontech) using *EcoR*I and *BamH*I, to generate plasmids expressing the fusion protein GFP-TEX13. Transient transfection of clones was achieved using Lipofectamine LTX transfection reagents and Lipofectamine 2000 (Invitrogen) according to the manufacturer's protocol. After 24 h of culture on coverslips, cells were fixed with 4% (v/v) paraformaldehyde and nuclei were labeled with Hoechst 33258 dye (Sigma). Samples were mounted on slides and visualized by microscopes (DMLB; Leica Microsystems and IX81; Olympus). Approximately 100 cells were scored per group. Transfection efficiency was 25.1%+/−5.1%.

## Results

### Identification of germ cell genes expressed in F9 cells

**Figure 1:**
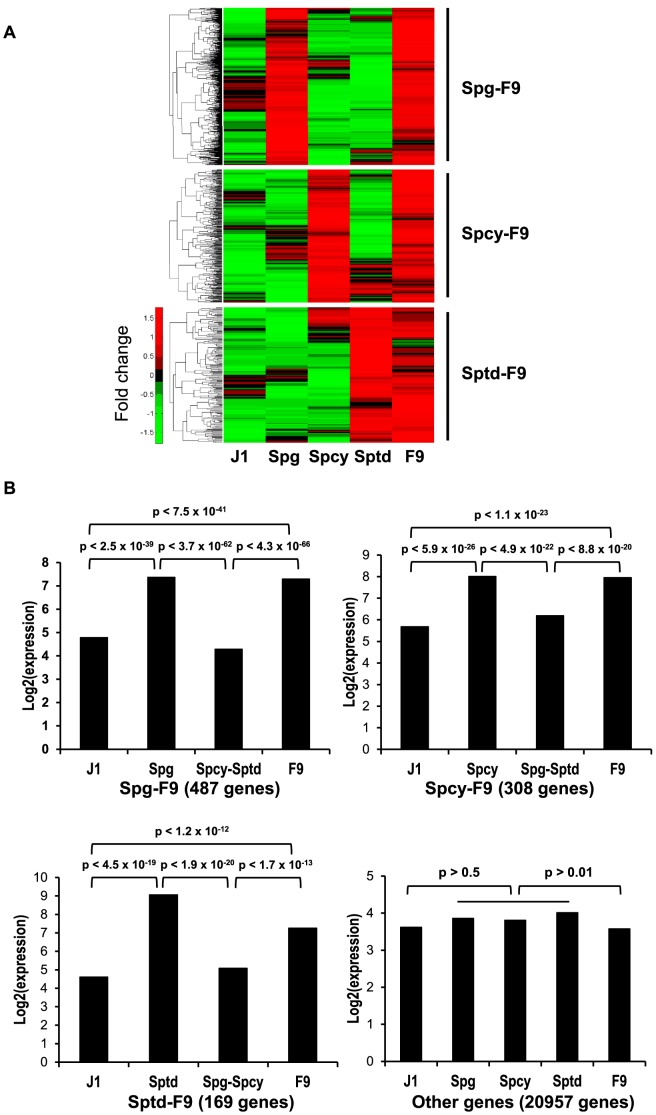
Microarray analysis of genes expressed stage-specifically in male germ cells and F9 cells. A. Heatmaps of the normalized gene expression profiles for each group. A total of 964 genes were expressed more than 1.5-fold higher in both a given germ cell stage and F9 cells compared to other germ cell stages and ESC. Clustering of each group was performed using hierarchical clustering. Up-regulated genes are indicated in red and down-regulated genes are shown in green. Abbreviations: J1, J1 embryonic stem cells; Spg, type B spermatogonia; Spcy, pachytene spermatocyte; Sptd, round spermatid; and F9, F9 cells. B. Mean expression level of all genes in each group. Differences between samples were validated using the Student's *t*-test. For simplification, the average expression levels between two germ cells with low expression levels were used. Significant differences were observed in the Spg-F9, Spcy-F9 and Sptd-F9 groups (*p*-value<10E-10).

To determine whether F9 cells transcribe genes that undergo stage-specific expression in male germ cells, we analyzed previously deposited microarray gene expression datasets representing type B spermatogonia (Spg), pachytene spermatocyte (Spcy), round spermatid (Sptd), F9 testicular teratoma cells (F9) and J1 embryonic stem cells (ESC). To avoid systematic variations in the analysis, we selected microarray datasets that were all produced using the Affymetrix Mouse Genome 430 2.0 array. Background adjustment of each array dataset was performed using GCRMA (GC Robust Multi-array Average) [16]. Quantile normalization was used to adjust the array data. Since F9 cells were isolated from a teratoma established by implanting an embryo with stem cell properties (pulipotency) in the testis, we used the ESC dataset as a negative control to reduce any influence from the stem cell properties of F9 cells. We selected genes that were up-regulated (≥1.5-fold change) in one of the germ cell stages and F9 cells relative to the other germ cell stages and ESC. We found that 487 genes were highly transcribed in spermatogonia and F9 cells (Spg-F9) (Figure 1A and Table S2), while 308 and 169 genes were up-regulated in spermatocytes and F9 cells (Spcy-F9) (Table S3) and spermatids and F9 cells (Sptd-F9), respectively (Table S4). Statistical analysis of each group indicated that the expression levels of the selected genes in the appropriate germ cell stage and F9 cells were significantly higher (*p*<10E-10), than in the other germ cell stages and ESC (Figure 1B). It should be noted that the Spg-F9 pattern had the largest number of genes among the three groups. Collectively, our *in silico* results demonstrated that numerous stage-specific germ cell genes (a total of 964 genes) are expressed in F9 cells.

**Figure 2:**
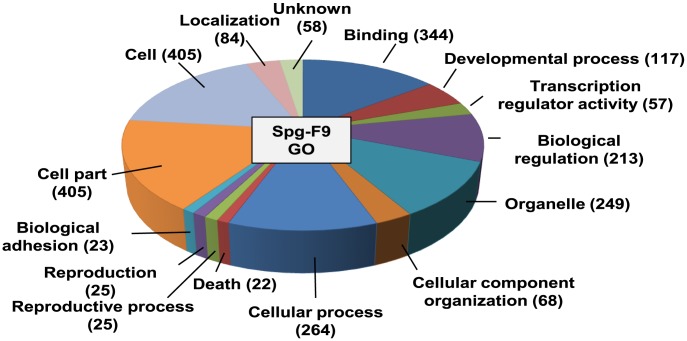
Enriched gene ontology (GO) terms in the spermatogonia-F9 group. A total of 487 genes in the Spg-F9 group were analyzed for the enrichment of GO terms using the DAVID functional annotation chart. Only significant GO terms are shown (*p*-value<0.05). The pie slices are proportional to the number of genes.

To further investigate the germ cell genes expressed in F9 cells, we performed gene ontology (GO) enrichment analysis using the DAVID functional annotation tool [17]. In this analysis, the enrichment score reflected the degree to which a GO term is overrepresented in the Spg-F9, Spcy-F9 or Sptd-F9 genes, compared to all genome-wide genes. Our results revealed that a number of GO terms were enriched in the Spg-F9 group (Figure 2), but not in the Spcy-F9 or Sptd-F9 groups. Using the criteria of a fold-enrichment score ≥1.5 and *p*<0.05, we found that the Spg-F9-enriched GO terms included transcription regulatory activity, developmental processes, death, biological adhesion, reproductive processes, and reproduction (Table 1). In particular, 57 genes encoding transcription factors were enriched under the GO term transcription regulatory activity; of them, 11 are known to function in germ cell proliferation, germ cell differentiation, and spermatogenesis (Table 2).

**Table 1:** Gene Ontology (GO) terms enriched in the Spg-F9 group.

GO term	# of genes	P-value	Fold enrichment
Transcription regulatory activity	57	9.90E-06	1.83
Developmental processes	117	1.70E-08	1.62
Death	22	0.03	1.61
Biological adhesion	23	0.03	1.56
Reproductive processes	25	0.03	1.53
Reproduction	25	0.03	1.52
Cellular component organization	68	0.0053	1.37
Localization	84	0.04	1.21
Biological regulation	213	4.99E-04	1.17
Binding	344	5.36E-10	1.17
Organelles	249	0.002	1.13
Cellular processes	264	0.098	1.08
Cell parts	405	0.04	1.02
Cells	405	0.04	1.02
Unknown	58	-	-

**Table 2:** Transcription factors related to spermatogenesis in Spg-F9 groups.

Entrez ID	Gene	Gene description	Reference
20475	*Six5*	Sine oculis-related homeobox 5	[22]
26423	*Nr5a1*	Nuclear receptor subfamily 5, group A, member 1	[23]
14605	*Tsc22d3*	TSC22 domain family, member 3	[24]
56484	*Foxo3*	Forkhead box O3	[25]
16478	*Jund*	Jun D proto-oncogene	[26]
18024	*Nfe2l2*	Nuclear factor, erythroid derived 2, like 2	[27]
12578	*Cdkn2a*	Cyclin-dependent kinase inhibitor 2A	[28]
14463	*Gata4*	GATA binding protein 4	[29]
18128	*Notch1*	Notch 1	[30]
50796	*Dmrt1*	Doublesex and mab-3 related transcription factor 1	[31]
20893	*Bhlhe40*	Basic helix-loop-helix family, member e40	[32]

Genes known as markers of spermatogonia, including *Dmrt1* (doublesex and mab-3 related transcription factor 1), *Stra8* (stimulated by retinoic acid gene 8) and *Gfra1* (glial cell line derived neurotrophic factor family receptor alpha 1) [18], were found to be expressed in F9 cells (Figure S1). In addition, various genes expressed in primordial germ cells, including *Prdm1* (PR domain containing 1, with ZNF domain) [19], *Prdm14* (PR domain containing 14) [20], and *Pou5f1* (POU domain, class 5, transcription factor 1) [21], are also predicted to be expressed in F9 cells (Figure S1).

### Identification of testis-specific or -predominant genes expressed in F9 cells

To identify testis-specific or -predominant (collectively called “testis-preferred”) genes among the germ cell genes found to be expressed in F9 cells, we calculated testis specificity using information from the UniGene database [33], which contains the EST (Expressed sequence tag) expression profile of a given gene in 47 tissues or organs based on transcription per million (TPM, indicating the normalized gene expression level). We identified nine genes as being putatively testis-specific or -predominant, using the criteria of testis specificity ≥50% or (for genes expressed in fewer than three tissues) 50%> testis specificity ≥30% (Table 3). The Spg-F9 group contained four testis-specific or -predominant genes: doublesex and mab-3 related transcription factor 1 (*Dmrt1*); testis expressed gene 13 (*Tex13*); nuclear receptor subfamily 5, group A, member 1 (*Nr5a1*); and stimulated by retinoic acid gene 8 (*Stra8*). The Spcy-F9 group contained a single such gene: Sp2 transcription factor (*Sp2*). The Sptd-F9 group contained four genes that were predicted to be specifically or predominantly expressed in testis: carboxypeptidase, vitellogenic-like (*Cpvl*); Fancd2 opposite strand (*Fancd2os*); tripartite motif family-like 1 (*Triml1*); and 1700061G19Rik. Interestingly, *Dmrt1* and *Stra8* are known to be involved in the initiation of meiosis [31], while *Nr5a1* is an important gene for reproductive differentiation [23]. The functions of the other listed genes are unknown.

**Table 3:** Testis-specific genes expressed in F9 cells.

Array pattern	Gene	UniGene ID	Testis-specificty (%)	Tissue
Spermatogonia-F9	*Dmrt1*	Mm.391208	96.88	Embryonic tissue, testis
	*Tex13*	Mm.193025	75	Embryonic tissue, testis
	*Nr5a1*	Mm.31387	51.79	Brain, embryonic tissue, fertilized ovum, lung, ovary, spleen, testis
	*Stra8*	Mm.5171	34.62	Embryonic tissue, testis
Spermatocyte-F9	*Sp2*	Mm.155547	62.07	Embryonic tissue, testis, thymus
Spermatid-F9	*Cpvl*	Mm.158654	100	Testis
	*Fancd2os*	Mm.72959	91.67	Kidney, testis
	1700061G19Rik	Mm.160106	85.16	Embryonic tissue, intestine, testis
	*Triml1*	Mm.64542	56	Embryonic tissue, extra embryonic tissue, testis

**Figure 3:**
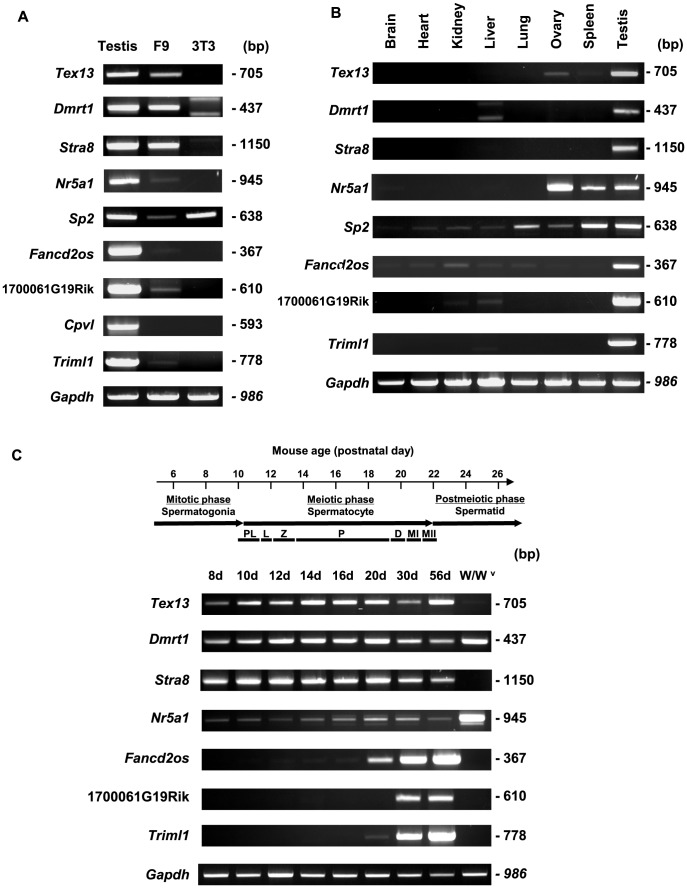
Tissue distribution and developmental expression patterns of germ cell-specific genes expressed in F9 cells. A. The expression patterns of nine genes predicted to be testis-specific or -predominant, as assessed in testis, F9 cells and NIH3T3 cells. Glyceraldehyde-3-phosphate dehydrogenase (*Gapdh*) was included as a loading control. Except for *Cpvl*, all of the genes were detected in both testis and F9 cells. It should be noted that the two bands for *Dmrt1* in NIH3T3 cells are non-specific, based on *in silico* investigation and an additional PCR analysis (data not shown). B. The tissue distributions of transcripts were assessed by RT-PCR analysis in various tissues of adult male mice. Complementary DNAs from various mouse tissues were amplified by PCR, with *Gapdh* included as a loading control. Seven genes were found to be testis-specific or -predominant. C. Developmental expression patterns during spermatogenesis. The stage-specific expression of the genes was determined from mouse testes on different days after birth (days 8, 10, 12, 14, 16, 20, 30, and 56). Abbreviations: *PL*, preleptotene; *L*, leptotene; *Z*, zygotene; *P*, pachytene; *D*, diplotene; *MI*, meiotic division I; and *MII*, meiotic division II. Complementary DNA from germ cell-lacking testes from *W/W^v^* mutant mice was also examined. Consistent with our microarray analysis, *Tex13*, *Stra8*, *Fancd2os*, 1700061G19Rik and *Triml1* were found to be germ cell-specific.

To confirm that these nine genes are expressed in F9 cells and show testis-specific or -predominant expression patterns, we performed *in vitro* expression analyses. RT-PCR analysis showed that, with the exception of *Cpvl*, all of the tested genes were transcribed both in testis and F9 cells (Figure 3A). NIH3T3 cells were used as a negative control for cell type-specific expression. The expression levels of *Tex13*, *Dmrt1*, and *Stra8* were similar between testis and F9 cells, whereas the other detected genes showed weaker expression in F9 cells than in testis. Next, we examined the tissue distribution of the eight genes confirmed to be expressed in F9 cells (Figure 3B). With the exception of *Nr5a1* and *Sp2*, all of the genes were expressed specifically or predominantly in testis, which was consistent with our *in silico* prediction. To investigate the developmental expression patterns of the testis-specific or -predominant genes, we performed RT-PCR using RNA from mouse testis samples obtained at different days after birth (Figure 3C). We hypothesized that if a given gene is transcribed in germ cells during spermatogenesis, the transcript will appear in the testis at a particular post-partum time point corresponding to a specific stage of spermatogenesis. The expression of all the four genes in the Spg-F9 group started at postnatal day 8, corresponding to spermatogonia. In contrast, *Fancd2os*, 1700061G19Rik and *Triml1* from the Sptd-F9 group were first expressed at postnatal days 20 or 30, corresponding to the late spermatocyte and round spermatid stages, respectively. Finally, we examined the germ cell-specific expression of the seven testis-specific or -predominant genes using the germ cell-lacking testes of *W/W^v^* (c-kit) mutant mice [34]. The transcripts corresponding to *Tex13*, *Stra8*, *Fancd2os*, 1700061G19Rik and *Triml1* were barely detected in the testes of mutant mice (Figure 3C), suggesting that these genes underwent germ cell-specific expression. Collectively, the results of our *in vitro* analyses of the *in silico*-selected genes demonstrated that six genes (*Tex13*, *Dmrt1*, *Stra8*, *Fancd2os*, 1700061G19Rik and *Triml1*) show testis-specific or- predominant expression in F9 cells. In particular, *Tex13* and *Stra8* show germ cell-preferred transcription with abundant expression in F9 cells.

### Promoter analysis of germ cell genes in F9 cells

**Figure 4:**
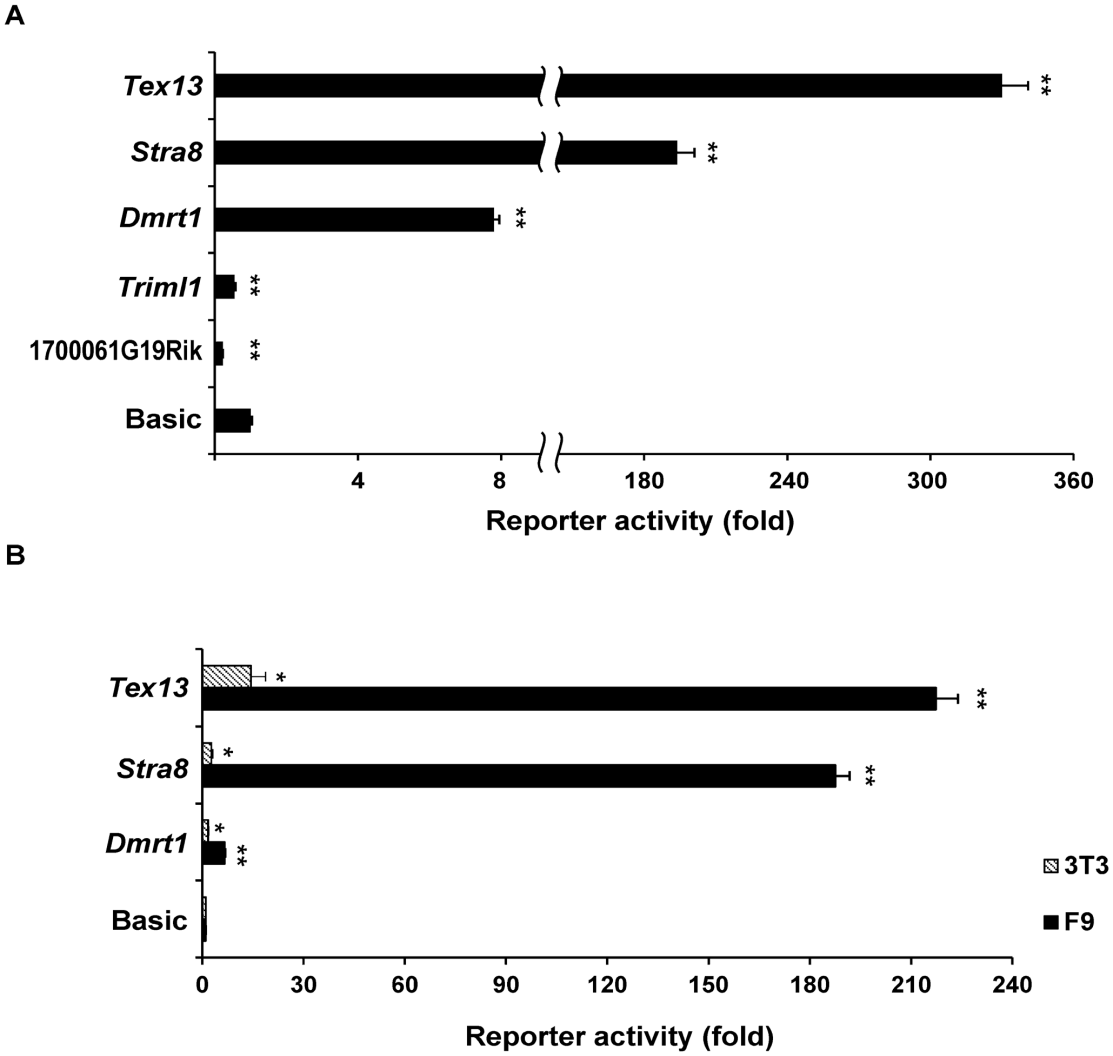
Promoter analysis of five germ cell-specific genes in F9 cells. A. Promoter-luciferase reporter analysis of five germ cell genes identified as being expressed in F9 cells. F9 cells were transfected with luciferase constructs containing 1.5-kb upstream regions spanning the transcription start site (TSS) (−1500 to +20 bp) of the indicated genes. The relative promoter activities are represented as the fold increase versus the expression from the promoter-less pGL3-Basic vector. The presented values represent the mean ± SD of three independent experiments. B. Promoter-luciferase reporter analysis of *Dmrt1*, *Stra8* and *Tex13* in NIH3T3 cells. Reporter constructs containing promoters were transiently transfected into NIH3T3 cells. The presented values represent the mean ± SD of three independent experiments. Statistical significance in A and B was determined by the Student's t-test; *p<0.05 and **p<0.01 versus pGL3-Basic.

To investigate the expression mechanisms governing the F9 cell-expressed germ cell genes, we performed transient transfection-reporter analyses of *Tex13*, *Dmrt1*, *Stra8*, 1700061G19Rik and *Triml1* (Figure 4A). We were unable to generate a reporter construct for *Fancd2os*. Upstream regions (∼1.5 kb) spanning the transcription start site (TSS) (−1500 to +20 bp) of each gene were cloned into the pGL3-Basic vector, and the dual luciferase assay was used to test for reporter activity in F9 cells (Figure 4A). We found that the upstream regions of the Spg-F9 genes, *Tex13*, *Stra8* and *Dmrt1*, exhibited significant promoter activities in F9 cells (Figure 4A). In particular, the promoters of *Tex13* and *Stra8* showed strikingly strong reporter activities (214- and 76-fold higher, respectively, than that driven by the vector alone). In contrast, and consistent with their weak expression levels in F9 cells (Figure 3A), the upstream regions of the Sptd-F9 genes (1700061G19Rik and *Triml1*) did not activate reporter gene expression in F9 cells. Their promoters rather suppressed the reporter activity of the control vector. We also monitored the reporter activity of the promoters of *Tex13*, *Stra8* and *Dmrt1* in NIH3T3 cells, and found that the promoters showed significantly lower activities in this somatic cell line compared to F9 cells (Figure 4B). Thus, our results indicate that F9 cells have active transcriptional machineries for the expression of spermatogonial genes, such as *Tex13*, *Stra8* and *Dmrt1*.

### Characterization of the *Tex13* promoter

**Figure 5:**
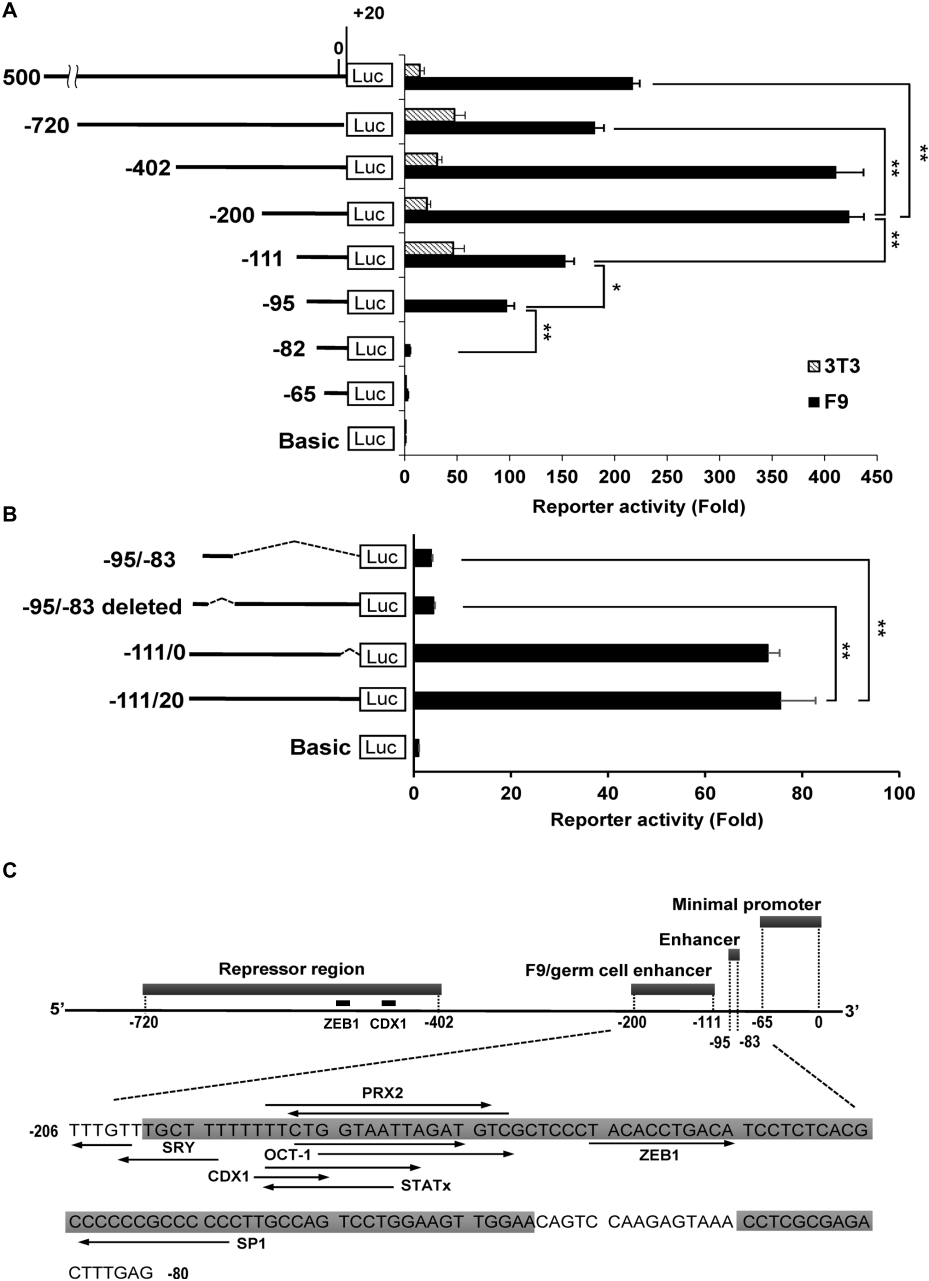
Promoter analysis of the sequence upstream of the mouse *Tex13* gene. A. Promoter activity of the murine *Tex13* 5′-flanking sequences. The *Tex13* upstream region used for the reporter assay is shown on the left. Various genomic regions were introduced into the promoter-less pGL3-Basic vector, and the reporter constructs were transiently transfected into F9 and NIH3T3 cells. Strong promoter activities were observed in F9 cells. The relative promoter activities are represented as the fold increase versus expression from pGL3-Basic. The presented values represent the mean ± SD of three independent experiments. Statistical significance was determined by the Student's t-test; *p<0.05, **p<0.01. All *p*-values versus pGL3-Basic were less than 0.01. B. Luciferase reporter analysis of the −95 bp to −83 bp region and the 0 to +20 bp region. The indicated deletion reporter constructs were prepared (left) and transiently transfected into F9 cells. The presented values represent the mean ± SD of three independent experiments. All *p*-values versus pGL3-Basic were less than 0.01. Statistical significance was determined by the Student's t-test; **p<0.01. C. Summary of relative luciferase activity from *Tex13* regulatory regions. The minimal promoter region and several enhancer/inhibitor regions are indicated upstream of the mouse *Tex13* gene. The locations of putative transcription factor-binding sites between −206 bp and −80 bp were predicted with the TFSEARCH program (arrow). The direction of each arrow indicates the orientation of the indicated putative element. The sequences of two enhancers are indicated (gray boxes). Binding sites for ZEB1 and CDX1, known to be transcriptional repressors, were predicted to be present in the −520 to −508 bp and −490 to −482 bp, respectively.

Previous functional and transcriptional studies on the *Dmrt1* and *Stra8* genes have been reported [35]–[40]. *Dmrt1* is essential for the differentiation of germ cells and Sertoli cells, where it is under the control of distinct transcriptional mechanisms. *Stra8* controls the switch from spermatogonial differentiation to meiosis, and its 400-bp promoter is known to direct gene expression in spermatogonial stem cells. In contrast, the expression patterns, expression mechanisms, and functions of *Tex13* have not previously been reported. Thus, we further investigated the promoter region of *Tex13* in F9 cells. To clarify the regions responsible for *Tex13* transcription, we cloned a series of 5′-nested deletions in the *Tex13* upstream region into the pGL3-Basic vector (−1500 to +20 bp, −720 to +20 bp, −402 to +20 bp, −200 to +20 bp, −111 to +20 bp, −95 to +20 bp, −82 to +20 bp and −65 to +20 bp) (Figure 5A). Transient transfection of F9 cells with these constructs revealed that the highest promoter activity (423-fold that of the vector control) was associated with the −200 to +20 bp region, whereas the lowest degree of significant activity (3-fold that of the vector control) was associated with the −65 to +20 bp region (Figure 5A). This suggests that the −65 to +20 bp region is a minimal promoter region for *Tex13* gene expression, and various additional repressive and enhancer regions are present in the −1500 to +20 bp region. Among the constructs harboring more than the −200 to +20 bp region, the −1500 to +20 bp and −720 to +20 bp regions, but not the −402 to +20 bp region, significantly inhibited the maximal activity of the −200 to +20 bp region. The degree of repression was similar between the −1500 to +20 bp and −720 to +20 bp regions, suggesting that the negative regulatory elements are present in the −720 to −402 bp region. All constructs smaller than the −200 to +20 bp region were found to exhibit gradual but significant reductions of promoter activity in F9 cells (Figure 3A). This suggests that the −200 to −65 bp region contains enhancer elements for *Tex13* transcription. We performed a similar promoter analysis in NIH3T3 cells. In contrast to our findings in F9 cells, the promoter activity of the −111 to +20 bp region was not reduced in these somatic cells, compared to the activity of the −200 to +20 bp region. Thus, our results suggest that the −200 to −111 bp region might be implicated in F9 cell- and germ cell-preferred expression. Finally, a 19-fold decrease in luciferase activity was observed in F9 cells transiently transfected with the construct spanning the −82 to +20 bp region of the *Tex13* promoter. This was the most dramatic reduction in reporter activity observed among the deletion constructs, indicating that a strong enhancer element is present in the region from −95 to −83 bp.

To further investigate this putative enhancer region, we generated constructs corresponding to the −95 to −83 bp region and the −110 to 0/+20 bp regions lacking the −95 to −83 bp sequence. F9 cells were transfected with these constructs or the intact −111 to 20 bp region (positive control), and the promoter activities of these constructs were compared (Figure 5B and 5C). We found that the −95 to −83 bp region alone exhibited very little promoter activity (Figure 5B), and deletion of the −95 to −83 bp region from the −111 to 0/+20 bp region dramatically decreased the promoter activity (8-fold reduction) (Figure 5C). These data confirm that the −95 to −83 bp region is a strong enhancer that is important for *Tex13* gene expression, and further indicate that the downstream sequence (the −82 to 0 bp region) is required for the full activity of the −95 to −83 bp region in the *Tex13* promoter.

We next performed sequence analysis of the *Tex13* promoter. The promoter lacks a typical TATA box. Analysis of the −200 to −83 bp region using the web-based tool, TFSEARH, revealed the presence of several potential transcription factor binding sites, including those for SRY (sex-determining region Y), CDX1 (caudal type homeobox 1), OCT-1 (POU domain, class 2, transcription factor 1), PRX2 (paired related homeobox 2), STATx (signal transducer and activator of transcription family), ZEB1 (zinc finger E-box binding homeobox 1) and SP1 (trans-acting transcription factor 1) (see Discussion). Figure 5C summarizes the data obtained from our promoter analyses.

### DNA methylation analysis of the *Tex13* promoter

**Figure 6:**
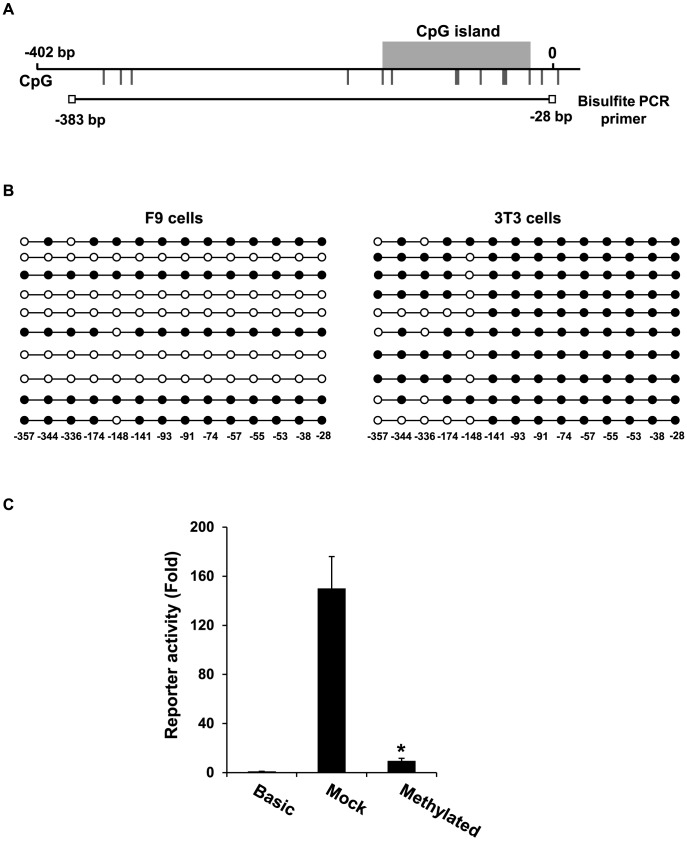
Methylation status of individual CpG sites in the *Tex13* promoter. A. The CpG islands of mouse *Tex13* were predicted with the MethPrimer program (http://www.urogene.org/ methprimer) and are indicated in gray. B. The DNA methylation status of individual CpG sites on the *Tex13* promoter was assessed by sodium bisulfite sequencing analysis. Genomic DNA was prepared from F9 and NIH3T3 cells. The black and white circles represent methylated and unmethylated CpGs, respectively. C. Effect of methylation on *Tex13* transcriptional activity. Luciferase constructs [pGL3-Basic and *Tex13* (−402/+20)] were *in vitro* methylated or mock-methylated with *Sss*I methyltransferase, and transfected into F9 cells. Firefly luciferase activity was assessed and normalized with respect to that of Renilla luciferase. Data are shown as relative fold increases compared with the results from mock-methylated pGL3-Basic. The presented values represent the mean ± SD of three independent experiments; **p<0.01 versus mock-methylated *Tex13* (−402/+20).

Although *Tex13* is not expressed in NIH3T3 cells (Figure 3A), the *Tex13* promoter exhibited low but significant reporter activity in this somatic cell line (Figure 4B and 5A). This suggests that an additional mechanism is involved in the germ cell-specific expression of *Tex13*. Since epigenetic regulation, such as DNA methylation, is closely related to gene expression [41], we examined whether *Tex13* undergoes epigenetic regulation. Sequence analysis of the proximal promoter of *Tex13* revealed that there are 14 CpG sites in the region from −383 bp to −28 bp (Figure 6A). We therefore used bisulfite sequencing to analyze the DNA methylation of this region in F9 and NIH3T3 cells. Our analysis revealed that 47% of these CpG sites were methylated in F9 cells. Interestingly, half of *Tex13* promoter in F9 cells were highly methylated, but the others were fully unmethylated (Figure 6B). In contrast, 85% of the sites were methylated in NIH3T3 cells, with nine CpG sites in the −141 to −28 bp region showing full methylation in NIH3T3 cells. This result suggests that DNA methylation of the −141 to −28 bp region of the *Tex13* promoter is associated with the expressional silencing of this gene in somatic cells but not in germ cells (i.e., germ cell-specific expression).

To determine whether DNA methylation of the *Tex13* promoter induces transcriptional silencing, we performed luciferase reporter analysis of the methylated promoter in F9 cells. We incubated a pGL3-Basic vector construct containing the −402 to +20 bp region of the *Tex13* promoter with *Sss*I (*M.SssI*) to methylate the cytosines of the 14 CpG sites. Promoter methylation was confirmed by methylation-sensitive restriction digest (data not shown). Compared to the mock-methylated control, the promoter activity of the *in vitro*-methylated −402 to +20 bp region was significantly suppressed in F9 cells (Figure 6C). Taken together, our results suggest that epigenetic regulation may help mediate the gene expression of *Tex13*.

### Expression and localization of the TEX13 protein

**Figure 7:**
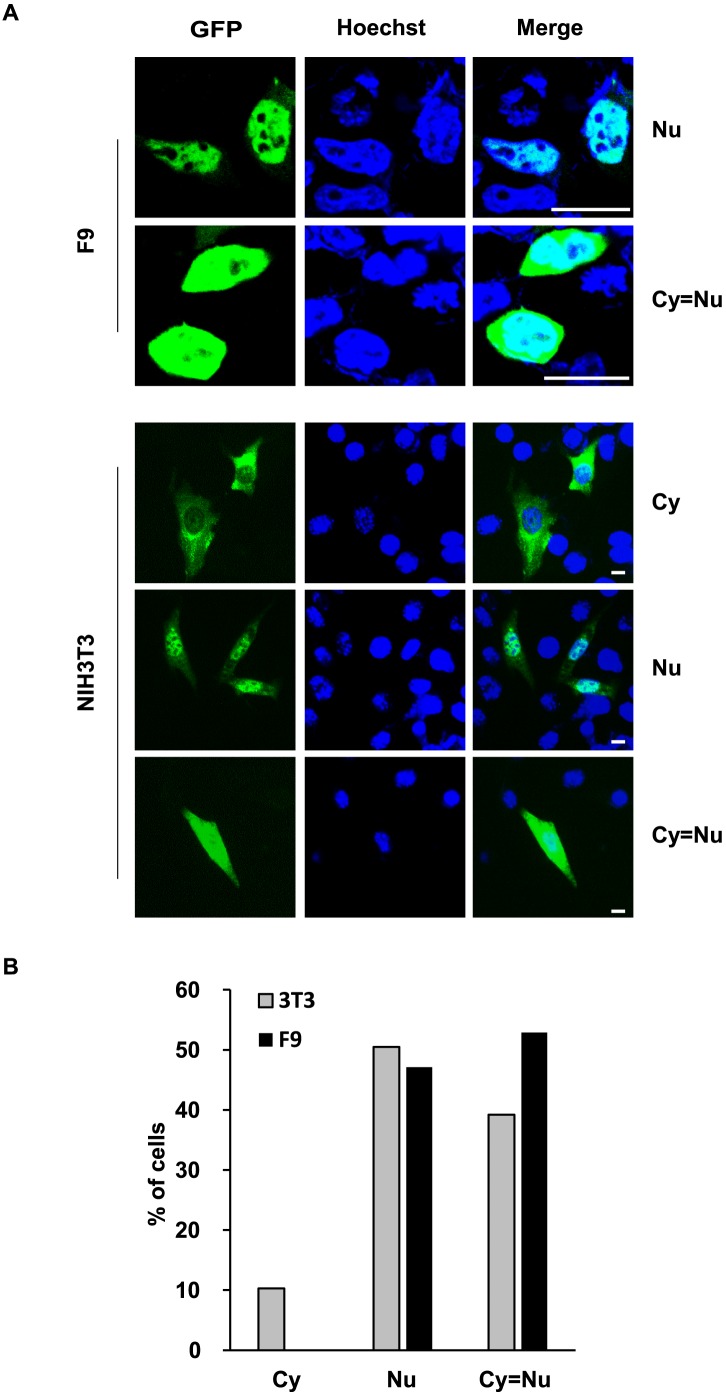
Localization of GFP-TEX13 in F9 cells and NIH 3T3 cells. A. Cells transiently expressing GFP-TEX13 fusion proteins were visualized under fluorescent light, and the protein location was determined. Hoechst 33258 dye (blue) was used to stain nuclei. GFP-TEX13 localization was heterogeneous. Scale bar, 20 µm. B. Quantification of the different patterns of TEX13 sub-cellular localization in F9 cells and NIH 3T3 cells. Scale bar, 20 µm. Nu, nuclear; Cy = Nu, diffuse; Cy, cytoplasmic.

*Tex13* is predicted to encode a 186-amino acid protein. To explore the characteristics of *Tex13* at the protein and cellular levels, we cloned its protein-coding sequence into the pEGFP-N2 vector and transiently expressed GFP-TEX13 proteins in F9 and NIH 3T3 cells (Figure 7A and 7B). In F9 cells, we observed that GFP-TEX13 localization was heterogeneous with the 47% of the transfected cells showing the protein exclusively in the nucleus and throughout the cytoplasm and nucleus (53%) (Figure 7A and 7B). A similar distribution was also observed in NIH 3T3 cells. The GFP-signal of TEX13 was predominantly located in the nucleus (51%) or evenly distributed both in cytoplasmic and nuclear compartments (39%). The rest of the transfected cells with the GFP-signal showed cytoplasmic localization (10%). Thus, our results suggest that the endogenous TEX13 protein can shuttle between nucleus and cytoplasm.

## Discussion

In this study, we investigated the gene expression profile of the F9 mouse testicular teratoma cell line versus male germ cells. Our *in silico* analyses revealed a total of 964 genes that are stage-specifically transcribed in male germ cells and also expressed in F9 cells. About the half of these genes were up-regulated in spermatogonia (Spg-F9 genes). Interestingly, the enriched GO terms were found only in this group; of them, the most prominently enriched GO term was transcription regulatory activity. A number of the Spg-F9 genes with this GO term are known to function in spermatogonial survival and spermatogenesis. Furthermore, spermatogonial markers and various genes expressed in primordial germ cells are also predicted to be expressed in F9 cells. Thus, F9 cells appear to transcribe genes that are expressed/active in both differentiated spermatogonia and pluripotent germ cell genes.

Genes that are expressed exclusively or predominantly in male germ cells are essential for spermatogenesis. Of the germ cell genes expressed in F9 cells, our *in silico* and *in vitro* analyses identified several as having specific or predominant expression in germ cells or testis. One of them, *Dmrt1*, encodes a protein that is essential in germ cells for the maintenance of embryonic germ cell identity and the development of juvenile spermatogonia [35], [36]. DMRT1 also inhibits meiosis in undifferentiated spermatogonia by blocking the retinoic acid-dependent robust expression of *Stra8* [31], which is required for meiotic initiation in both sexes. In germ cells lacking STRA8, the mitotic development of germ cells is normal, but they fail to go through meiotic initiation or progression [38], [39]. *Tex13* is an X-linked gene expressed in spermatogonia [42], but little was known about the expression mechanism or function of this gene prior to this study. *Dmrt1*, *Stra8* and *Tex13* are preferentially expressed in spermatogonia compared to the other germ cell stages, whereas *Triml1*, *Fancd2os* and 1700061G19Rik are expressed majorly in spermatids. *Triml1* is involved in the ubiquitin pathway [43], whereas the expression patterns and functions of *Fancd2os* and 1700061G19Rik are not yet known. However, these genes are expressed very weakly in F9 cells. Considering its expression pattern and unexplored function, we therefore decided to explore the transcription and function of *Tex13*, using F9 cells as a model system. Studies on germ cell-specific and -predominant genes have been limited by the lack of stable cell lines expressing such genes. F9 cells could therefore be an alternative tool for investigating the transcriptional regulation of such genes. Using F9 cells, we found that the 1.5-kb regions upstream of *Dmrt1*, *Stra8* and *Tex13* exhibited strong reporter activities. Furthermore, we showed for the first time that the *Tex13* promoter is located −200 to 0 bp upstream of the gene. The short sequence spanning the −95 to −83 bp region is responsible for strong promoter activity, but requires the downstream sequences for its activity. Since no known transcription factor is predicted to interact with this region, we speculate that *Tex13* transcription might involve the binding of an unknown transcription factor(s) to the −95 to −83 bp region to form a complex with other transcription factors that interact with the downstream region. We also discovered an additional critical region (−200 to −111 bp) in the proximal promoter of *Tex13*. Our data suggest that this region is implicated in the F9 and germ cell-preferred transcription of the gene. The SRY, CDX1, OCT-1, PRX2, STAT, ZEB1 and SP1 transcription factors are predicted to bind to the region. Of them, PRX2 is not expressed in spermatogonia [44]; ZEB1 and CDX1 are transcriptional repressors [45], [46]. Putative binding sites of these repressors are also located in the repressor region −720 to −402 bp. OCT1 is a ubiquitous transcriptional activator [47] that also reportedly functions as a transcriptional repressor in certain genes [48]. Some of the STAT family members are important for PGC formation and spermatogenesis [49], [50], while SP1 is known to mediate transcriptional activation and repression in male germ cells [51]. The −200 to −111 bp region associated with these transcription factors could therefore be involved in the transcriptional activation or repression of *Tex13* in different cell types. Further studies are needed to determine whether this transcriptional regulation is related to the germ cell-specific expression of *Tex13*.

Methylation of cytosines at CpG dinucleotides is a well-defined mechanism of epigenetic gene silencing in mammals [41], and promoter methylation has been associated with the testis- or cell type-specific expression of certain genes during spermatogenesis [52], [53]. Here, our transient transfection-reporter analysis revealed that the promoter region of *Tex13* showed weak but significant activities in NIH3T3 cells, despite the lack of endogenous *Tex13* gene expression in these cells. This prompted us to investigate whether *Tex13* expression is regulated by DNA methylation. Bisulfite sequencing revealed that the *Tex13* promoter is hypermethylated in NIH3T3 cells, but not in F9 cells. We further found that *in vitro* methylation of the promoter region significantly inhibited the reporter activity. Thus, differential promoter methylation may contribute to the testis- and germ cell-predominant expression of *Tex13*. Interestingly, half of the F9 genomic DNA samples exhibited hypermethylation in the *Tex13* proximal promoter, while the others were completely demethylated. In relation to this, it is noteworthy that embryonal carcinomas and their derived cell lines, including F9 cells, are composed of an undifferentiated stem cell population and a differentiated tumor cell population. The X chromosome is methylated by X-inactive specific transcript (XIST) in differentiated germ cell tumors but not in undifferentiated germ cell tumors, as shown in the case of the androgen receptor gene [54].

Finally, subcellular localization is a critical characteristic used to elucidate protein functions. We found that the localization of GFP-TEX13 was heterogeneous in the cytoplasmic and nuclear compartments, suggesting that endogenous TEX13 can shuttle between the two compartments. The N-terminal region of TEX13 was predicted from sequence analysis to contain a putative nuclear localization sequence (data not shown). Nuclear-cytoplasmic shuttling plays an important role in regulating the activity of several proteins involved in cell cycle progression and proliferation, and signal transduction [55]. It should be noted that the properties of *Tex13*, including the subcellular localization of its encoded protein, are similar to those of *Stra8*. It has been reported that STRA8 associates with DNA and possesses transcriptional activity [56], and a mouse knockout study found that STRA8 is crucial for meiotic progression during spermatogenesis [38], [39]. Based on our findings, we speculate that TEX13 may act as a transcription factor or cooperate with transcription factors to regulate the expression levels of genes involved in early germ cell development. A previous proteomic study reported the presence of the TEX13 protein in early male germ cells [57]. Further investigations are needed to determine the precise function of TEX13 in male germ cell development.

In conclusion, we herein report a comprehensive analysis of genes transcribed in both F9 cells and male germ cells. Our data suggest that F9 cells can transcribe genes that exhibit stage-specific expression in early germ cells. In particular, we identified a F9 cell-expressed germ cell-specific gene, *Tex13*, and used the cell line to characterize its promoter and potential epigenetic regulation. Finally, we found that TEX13 is a potential nucleocytoplasmic shuttling protein. Our results collectively establish a basis for future investigations into the transcriptional mechanism of *Tex13* and the nuclear function of its encoded protein.

## Supporting Information

Figure S1 **Expression profile of genes known to be transcribed in spermatogonia and primordial germ cells.** Heatmap of the normalized gene expression profiles of genes is shown. Up-regulated genes are indicated in red and down-regulated genes are shown in green. Abbreviations: J1, J1 embryonic stem cells; Spg, type B spermatogonia; Spcy, pachytene spermatocyte; Sptd, round spermatid; and F9, F9 cells. Genes: Plzf, promyelocytic leukemia zinc finger ortholog; Dmrt1, doublesex and mab-3 related transcription factor 1; Stra8, stimulated by retinoic acid gene 8; Gfra1, glial cell line derived neurotrophic factor family receptor alpha 1; Klf2, kruppel-like factor 2; Lin28, RNA-binding protein LIN-28; Nanog, nanog homeobox; Sox2, SRY (sex determining region Y)-box 2; Pou5f1, POU domain, class 5, transcription factor 1; Tcfap2c, transcription factor AP-2, gamma; Prdm14, PR domain containing 14; Prdm1, PR domain containing 1, with ZNF domain.
(PDF)

Table S1 **Oligonucleotides used for plasmid construction, site-directed mutagenesis and bisulfite sequencing.**
(XLSX)

Table S2 **Summary of gene list in Spg-F9.**
(XLSX)

Table S3 **Summary of gene list in Spcy-F9.**
(XLSX)

Table S4 **Summary of gene list in Sptd-F9.**
(XLSX)
