# Supplementary material for: Identification and Characterization of Germ Cell Genes Expressed in the F9 Testicular Teratoma Stem Cell Line
Source: PLoS One. 2014 Aug 25;9(8):e103837. doi: 10.1371/journal.pone.0103837 (PMC4143169; doi:10.1371/journal.pone.0103837)
Supplement: Figure S1 — Expression profile of genes known to be transcribed in spermatogonia and primordial germ cells. Heatmap of the normalized gene expression profiles of genes is shown. Up-regulated genes are indicated in red and down-regulated genes are shown in green. Abbreviations: J1, J1 embryonic stem cells; Spg, type B spermatogonia; Spcy, pachytene spermatocyte; Sptd, round spermatid; and F9, F9 cells. Genes: Plzf, promyelocytic leukemia zinc finger ortholog; Dmrt1, doublesex and mab-3 related transcription factor 1; Stra8, stimulated by retinoic acid gene 8; Gfra1, glial cell line derived neurotrophic factor family receptor alpha 1; Klf2, kruppel-like factor 2; Lin28, RNA-binding protein LIN-28; Nanog, nanog homeobox; Sox2, SRY (sex determining region Y)-box 2; Pou5f1, POU domain, class 5, transcription factor 1; Tcfap2c, transcription factor AP-2, gamma; Prdm14, PR domain containing 14; Prdm1, PR domain containing 1, with ZNF domain. (PDF) [file pone.0103837.s001.pdf]

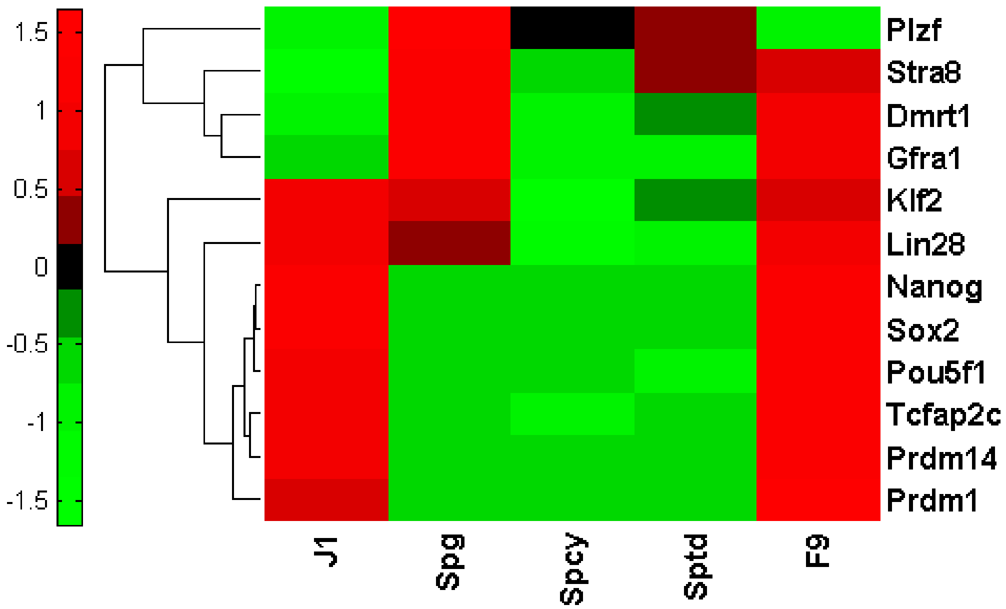

Figure S1. Expression profile of genes known to be transcribed in spermatogonia and primordial germ cells. Heatmap of the normalized gene expression profiles of genes is shown. Up-regulated genes are indicated in red and down-regulated genes are shown in green. Abbreviations: J1, J1 embryonic stem cells; Spg, type B spermatogonia; Spcy, pachytene spermatocyte; Sptd, round spermatid; and F9, F9 cells. Genes: *Plzf*, promyelocytic leukemia zinc finger ortholog; *Dmrt1*, doublesex and mab-3 related transcription factor 1; *Stra8*, stimulated by retinoic acid gene 8; *Gfra1*, glial cell line derived neurotrophic factor family receptor alpha 1; *Klf2*, kruppel-like factor 2; *Lin28*, RNA-binding protein LIN-28; *Nanog*, nanog homeobox; *Sox2*, SRY (sex determining region Y)-box 2; *Pou5f1*, POU domain, class 5, transcription factor 1; *Tcfap2c*, transcription factor AP-2, gamma; *Prdm14*, PR domain containing 14; *Prdm1*, PR domain containing 1, with ZNF domain.
